# Supplementary material for: Hippocampal place cell sequences differ during correct and error trials in a spatial memory task
Source: Nat Commun. 2021 Jun 7;12:3373. doi: 10.1038/s41467-021-23765-x (PMC8185092; doi:10.1038/s41467-021-23765-x)
Supplement: Supplementary file 1 — Supplementary Information [file 41467_2021_23765_MOESM1_ESM.pdf]

Supplementary Information for

Hippocampal Place Cell Sequences Differ during Correct and Error Trials in a Spatial Memory Task

SUPPLEMENTARY FIGURES

Supplementary Figure 1. Over-representation of reward location after learning and accuracy of decoding.

a, Normalized firing rates of CA1 place cells recorded from all sessions are shown for pre-running trials. Place cells were sorted by their peak firing positions after pre-running trials (i.e., during sample, test, and post-test trials). Position was aligned to the reward location on the horizontal axis (i.e., position 0). **b**, Proportion of total cells that exceeded a firing rate threshold of 0.5 Hz across aligned positions in pre-running trials. **c-d**, Same as **a-b** but for sample, test, and post-test trials. **e**, A cumulative graph of decoding errors for each recording session. Each gray trace indicates an individual recording session. **f**, A mean confusion matrix averaged across all recording sessions.

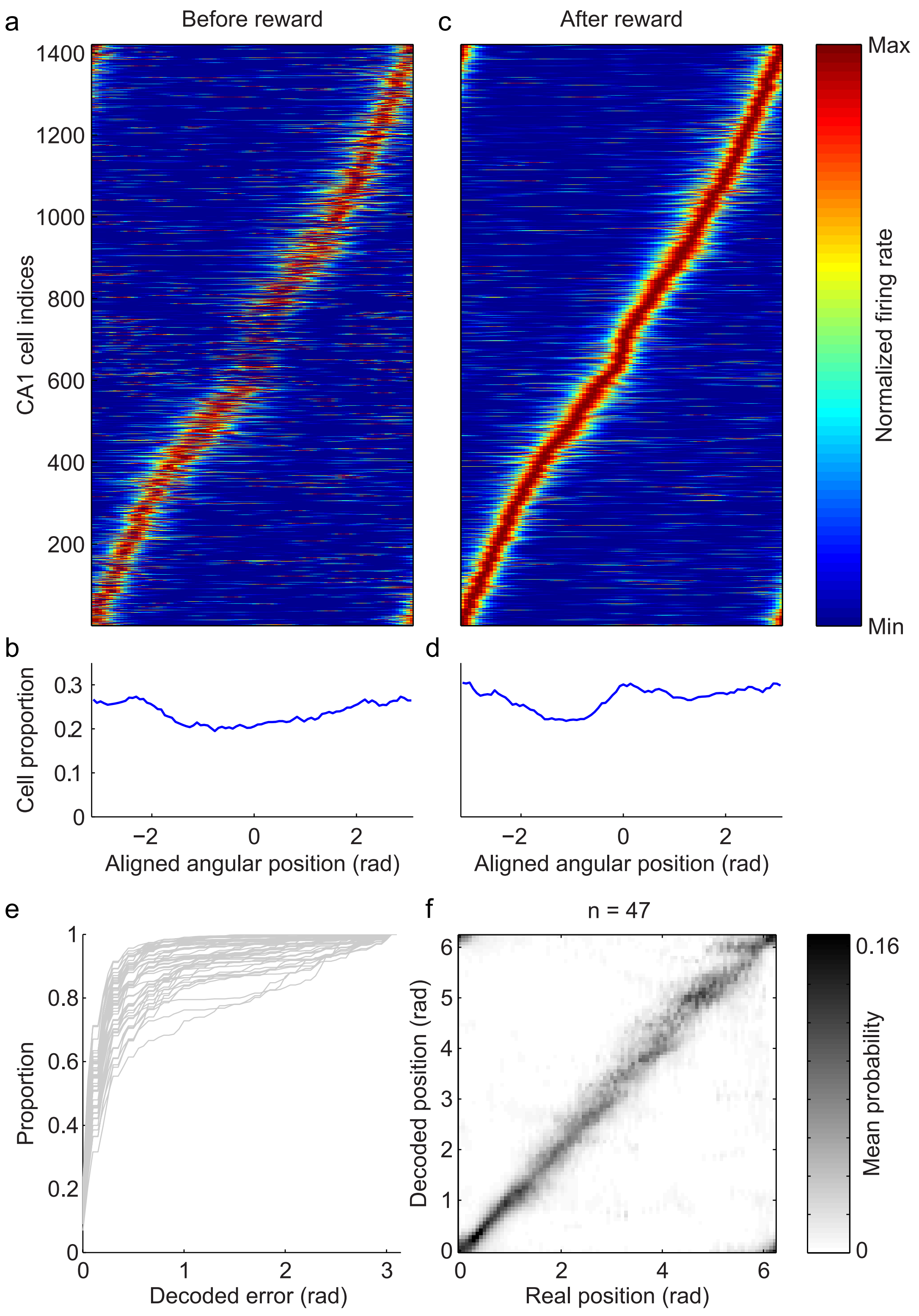

Supplementary Figure 1

Supplementary Figure 2. Examples of posterior probability distributions from test trials with different types of errors

a-b, Same as **Fig. 3 a-b** but showing a different trial pair example from a different recording session. In this example, the rat stopped one location before the correct reward location in the error test trial (“Error (-1) test trial”). **c-d**, Same as **a-b** but showing a different trial pair example from another recording session in a different rat. In this example, the rat stopped one location later than the correct goal location in the error test trial (“Error (+1) test trial”).

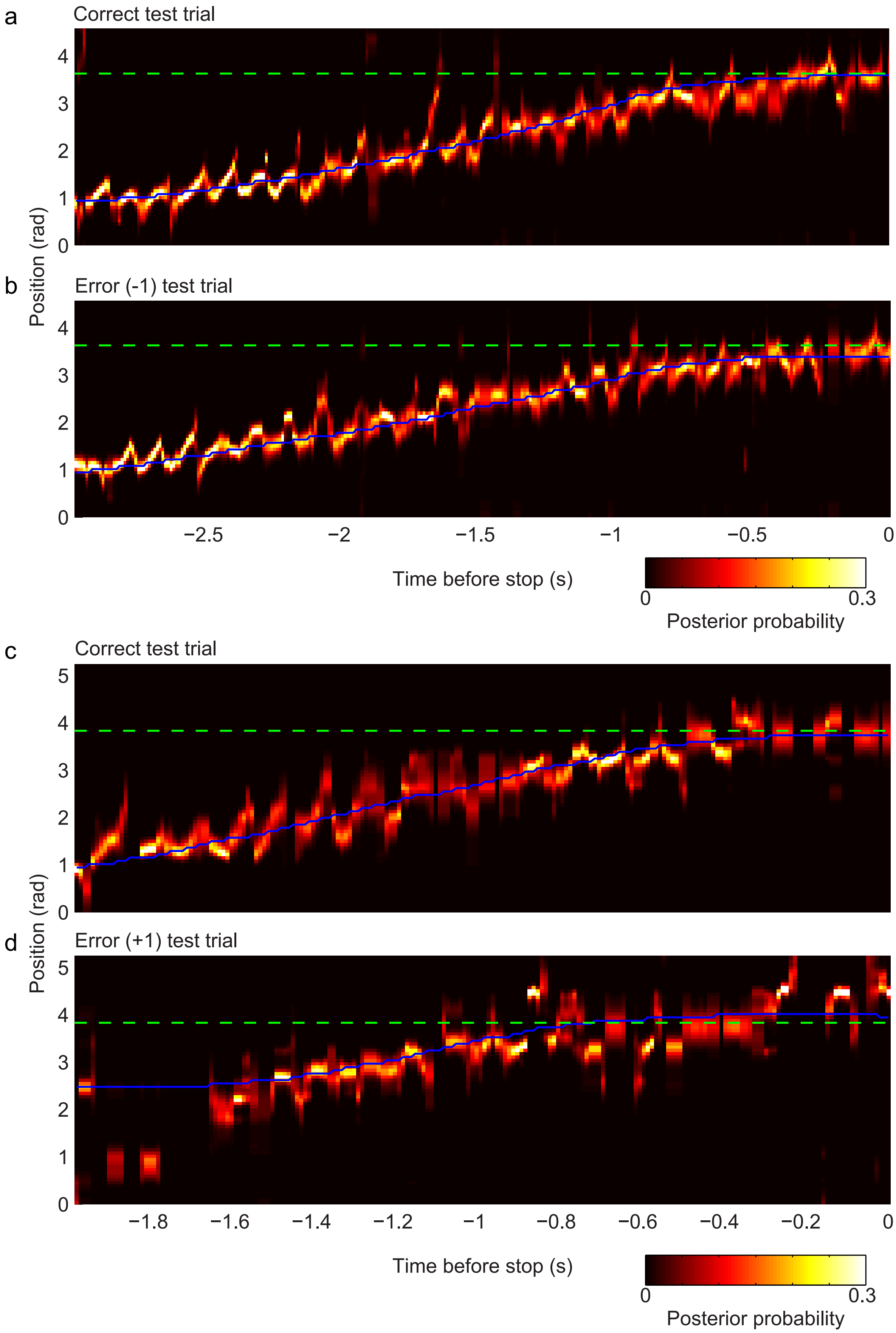

Supplementary Figure 2

Supplementary Figure 3. Running speeds during the approach to the stop location

a, Running speed estimates during the sample phase of the task are shown across location numbers as rats approached their stop location (indicated as location 0; the stop location was the same as the correct goal location for correct trials and was the incorrect stop location for error trials). Correct trials (n=220) are shown in red and error trials (n=124) are shown in black. **b,** Same as **a** but for the test phase. Data are presented as mean $\pm$ 95% bootstrapped confidence interval.

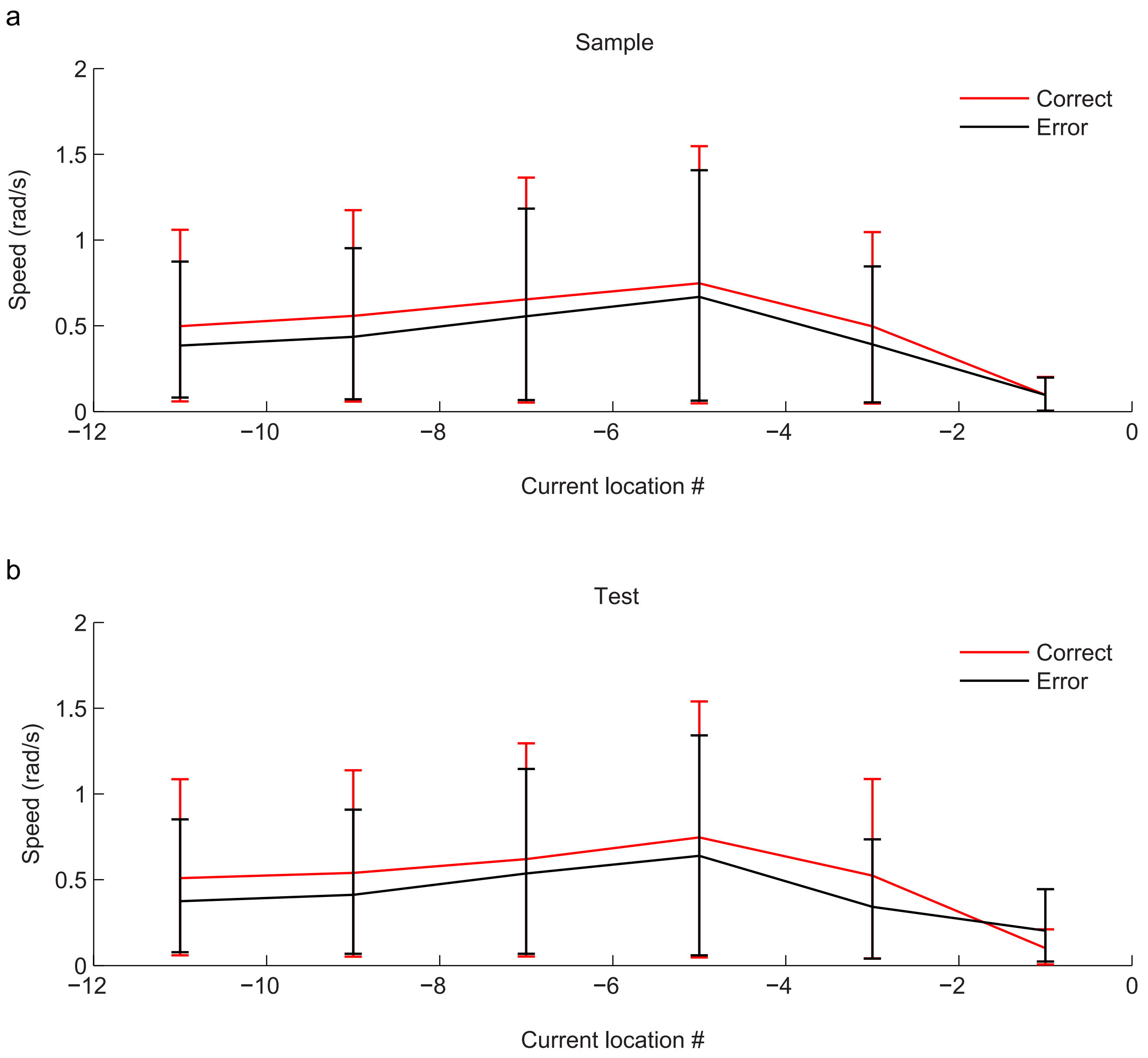

Supplementary Figure 3

Supplementary Figure 4. Distances ("x-span") and durations ("t-span") of sequences during correct and error trials. a-c, Mean x-span (**a**), mean relative x-span (**b**), and mean t-span (**c**) of lines that were fit to place cell sequences are shown across locations as rats approached their stop location during the test phase of trials (ANOVA, main effect of trial type (i.e., correct vs. error) on (**a**) x-span: $F(1,2826) = 13.9$, $p = 2.0 \times 10^{-4}$, (**b**) relative x-span: $F(1,2826) = 10.9$, $p = 9.5 \times 10^{-4}$ and (**c**) t-span: $F(1,2826) = 1.3$, $p = 0.2$; n = 1879 positive sequences detected from correct test trials and n = 959 positive sequences detected from error test trials). The relative x-span was calculated to control for the actual moving distance caused by varying running speeds during each sequence. The relative x-span was calculated as the original x-span minus moving distance. **d-f**, Same as **a-c** but for place cell sequences during the sample phase (ANOVA, main effect of trial type on (**a**) x-span: $F(1,2838) = 12.4$, $p = 4.3 \times 10^{-4}$, (**b**) relative x-span: $F(1,2838) = 11.6$, $p = 6.8 \times 10^{-4}$ and (**c**) t-span: $F(1,2838) = 0.9$, $p = 0.4$; n = 1818 positive sequences detected from correct sample trials and n = 1032 positive sequences detected from error sample trials). The measurements are shown plotted for the center of each location bin (i.e., locations -12 to -10 plotted at location -11, locations -10 to -8 plotted at location -9, etc.). Data are presented as mean ± error bars. Error bars indicate 95% bootstrapped confidence intervals.

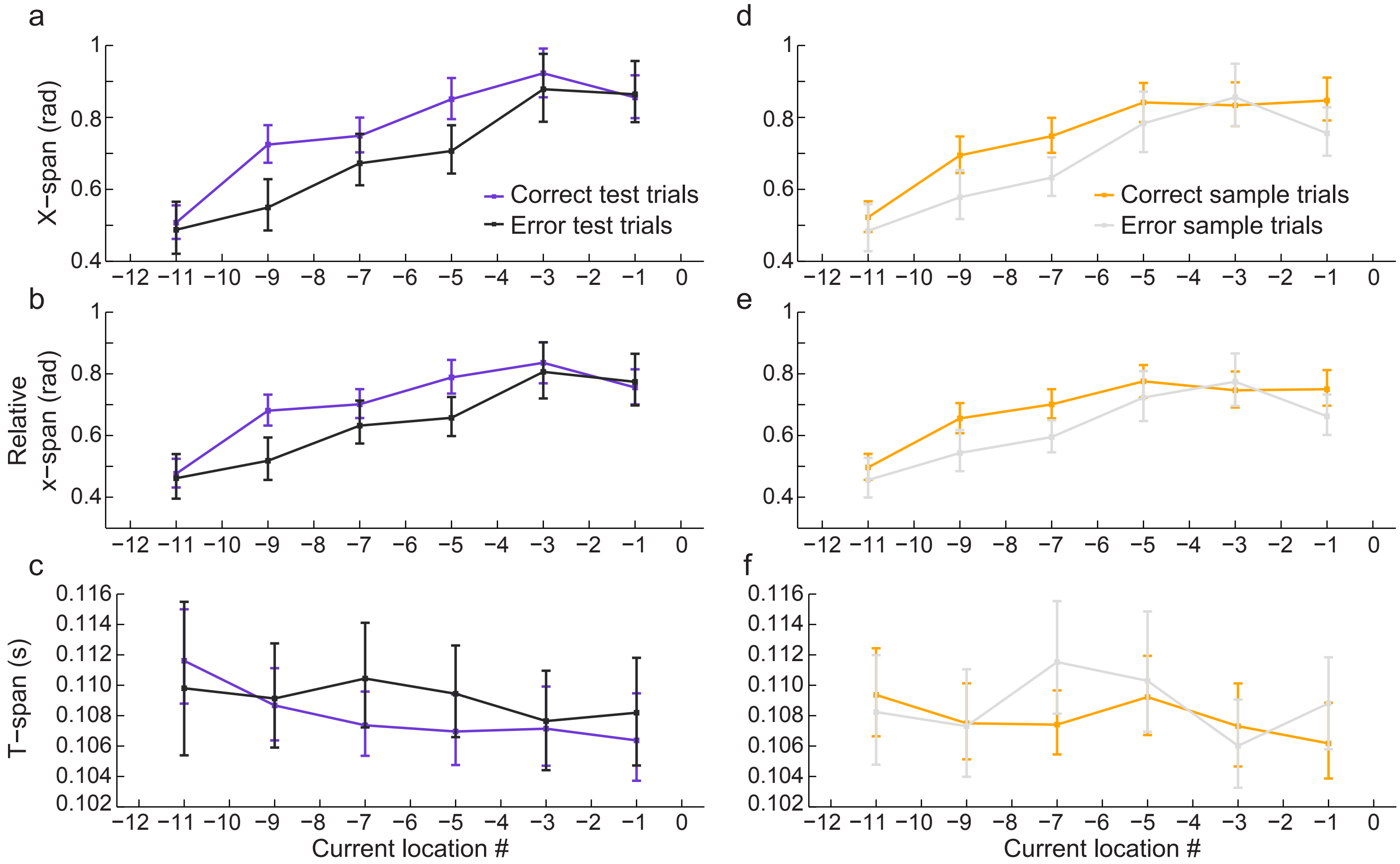

Supplementary Figure 4

Supplementary Figure 5. Gamma phase modulation of place cell spikes was similar between different trial types.

In our earlier work, we showed that spikes at successive slow gamma phases tended to code sequences of locations[1]. We hypothesized that this slow gamma phase coding is important for activating compressed representations of upcoming spatial trajectories and would be deficient during errors in a spatial memory task. To test this hypothesis, we compared distributions of slow gamma phases of spike times as rats approached their stop locations during correct and error trials. Based on our earlier findings[1], we also hypothesized that fast gamma phase-locking of spikes promotes real-time memory encoding. Thus, we also compared distributions of fast gamma phases of spikes as rats approached their stop locations during correct and error trials. **a**, Probability distributions of slow gamma phases of spikes across slow gamma cycles (Cycle -1, 0, 1) within place cell sequences for each trial type. Slow gamma phases of spikes shifted systematically across successive slow gamma cycles in both correct and error trials for both sample and test phases of the task. **b**, Probability distributions of fast gamma phases of spikes across successive fast gamma cycles (Cycle -2, -1, 0, 1, 2) within place cell sequences for each trial type. Fast gamma phase distributions remained similar across successive fast gamma cycles and across trial types. **c**, Circular-linear regression between slow gamma phases of spikes and cycle number for each trial type indicate that slow gamma phases of place cell spikes shifted across successive gamma cycles during both correct and error trials. **d**, Mean vector lengths of slow gamma phases of place cell spikes for each trial type, pooled across all slow gamma cycles. **e,** Mean vector lengths of fast gamma phases of place cell spikes for each trial type,

pooled across all fast gamma cycles. Mean vector lengths of fast gamma phase distributions were similar across trial types, indicating that fast gamma phase-locking did not significantly differ across correct and error trials. **f,** Circular-linear regression between fast gamma phases of spikes and cycle number for each trial type. Black dashed lines in **c-f** mark 95% confidence intervals of a null distribution generated by shuffling trial types.

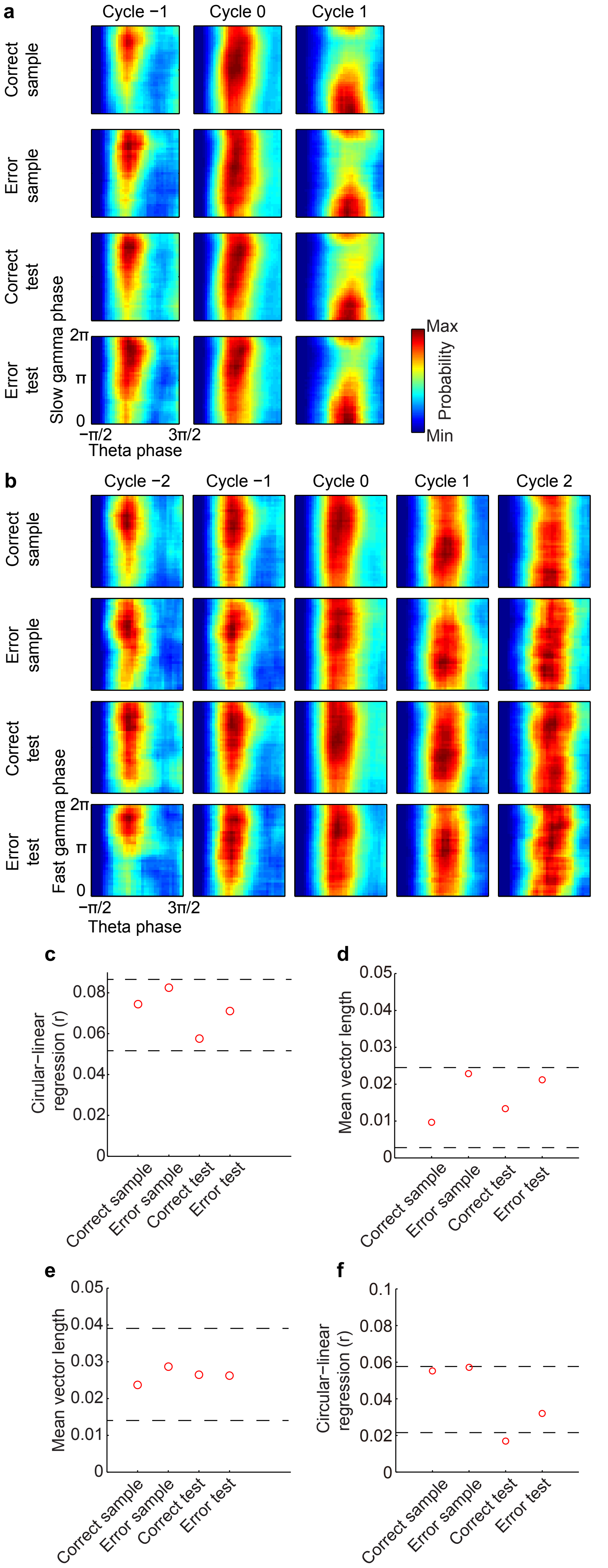

Supplementary Figure 5

Supplementary Figure 6. No difference in gamma power was observed between trial types as rats approached a stop location.

a, Example of detected slow and fast gamma events during sample and test phases of correct and error trials. Shown are examples of raw LFP recordings with power in the 97th, 98th, and 99th percentiles for each gamma type. **b**, Mean normalized power averaged across the theta frequency band (6-12 Hz) for sample trials (far left panel), test trials (2nd panel), downsampled sample trials (3rd panel), and downsampled test trials (far right panel). Color labels indicate correct, downsampled correct ("Correct$_{ds}$"), and error trials (dark green, light green, and black, respectively). Shaded area indicates 95% bootstrapped confidence intervals. **c-d**, Same as **b** but for mean normalized power averaged across the slow gamma ("SG", 25-55 Hz) and fast gamma ("FG", 60-100 Hz) frequency bands, respectively. **e**, Relationship between place cell sequence slopes and gamma power for each trial type. Slopes and gamma power estimates for each place cell sequence were ranked and normalized for each trial type. Slow (blue) and fast (magenta) gamma power were plotted separately against sequence slopes. Data are presented as mean ±error bars. Error bars indicate 95% bootstrapped confidence intervals (n=220 and 124 for correct and error trials respectively).

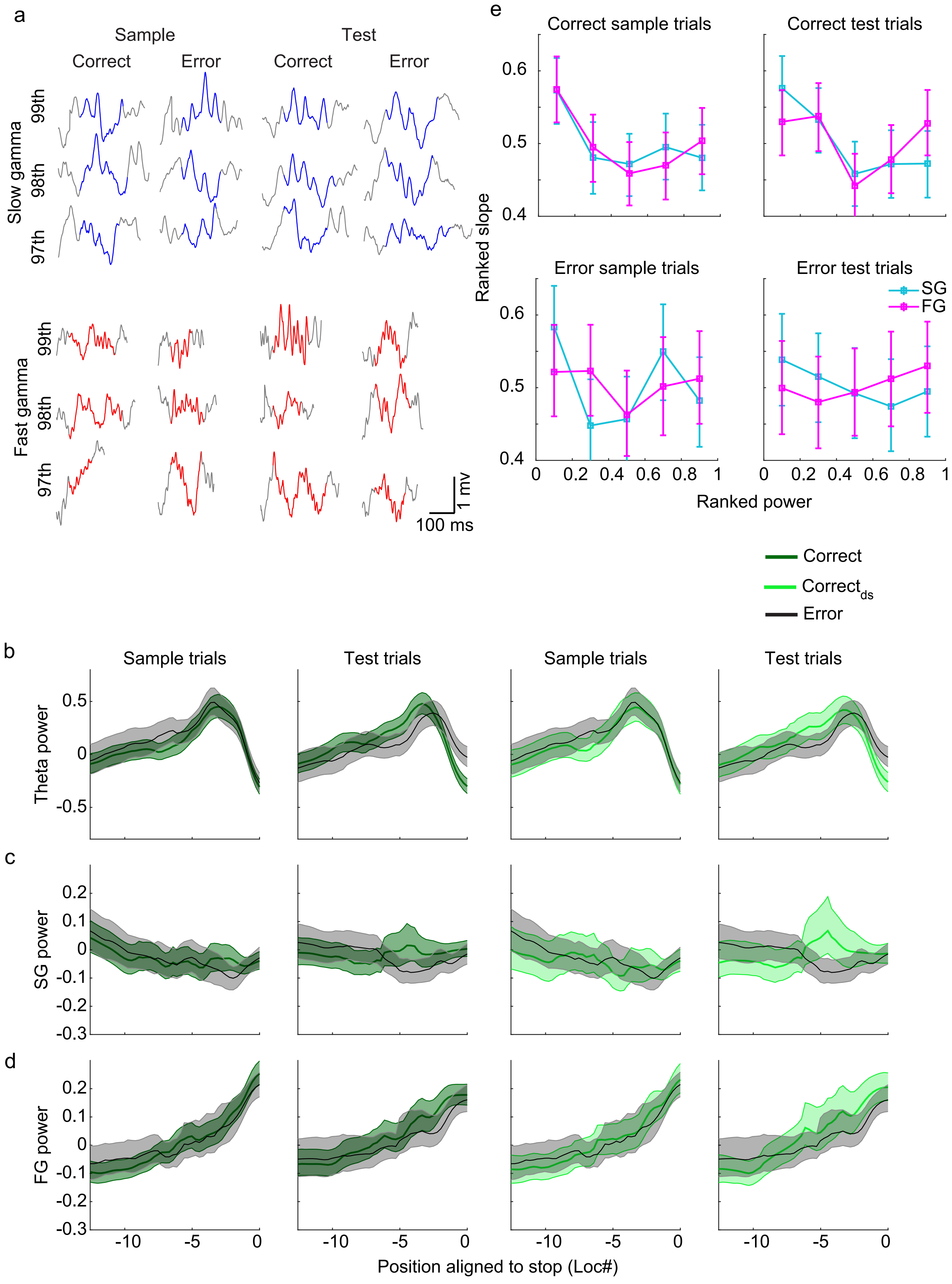

Supplementary Figure 6

Supplementary Figure 7. Place cell sequences with negative slopes detected during correct and error trials.

a-c, Same as **Fig. 3 c-e** but for sequences with negative slopes (ANOVA, significant main effects of trial type: $F(1,1546) = 13.2$, $p = 2.9 \times 10^{-4}$ for test phase, n = 1016 negative sequences detected from correct test trials and n = 542 negative sequences detected from error test trials). **d-f**, Same as **a-c** but for negative slope sequences from the sample phase (ANOVA, significant main effects of trial type: $F(1,1599) = 7.3$, $p = 0.007$ for sample phase, n = 1036 negative sequences detected from correct sample trials and n = 575 negative sequences detected from error sample trials).

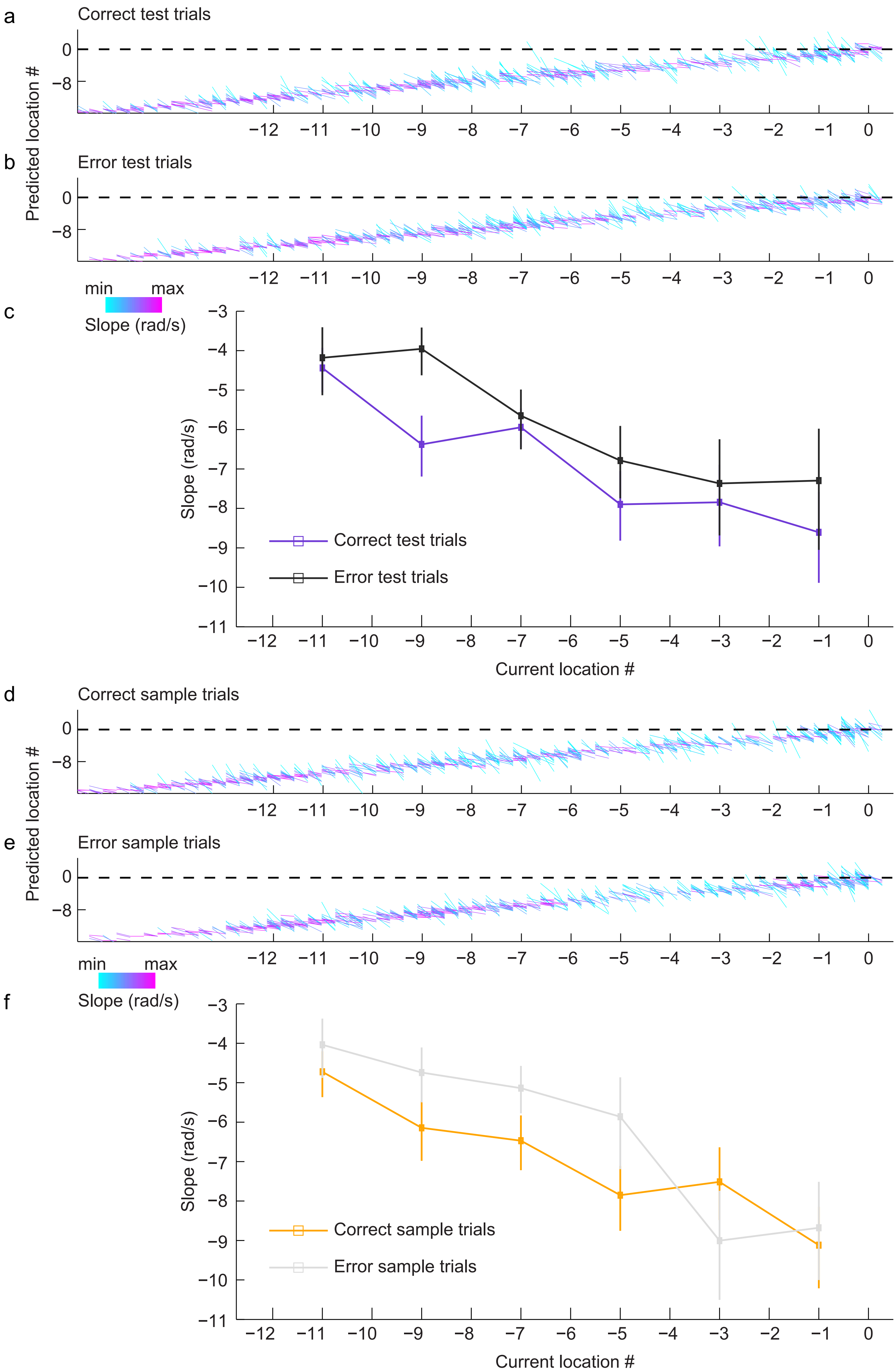

Supplementary Figure 7

Supplementary Figure 8. Forward and reverse replay fidelity was similar between rest periods from correct and error trials.

Solid lines indicate distributions of r^2 values for forward (left panel) and reverse (right panel) replay events for all detected SWR events in correct (red) and error (black) trials. Shaded error bars indicate 95% bootstrapped confidence intervals.

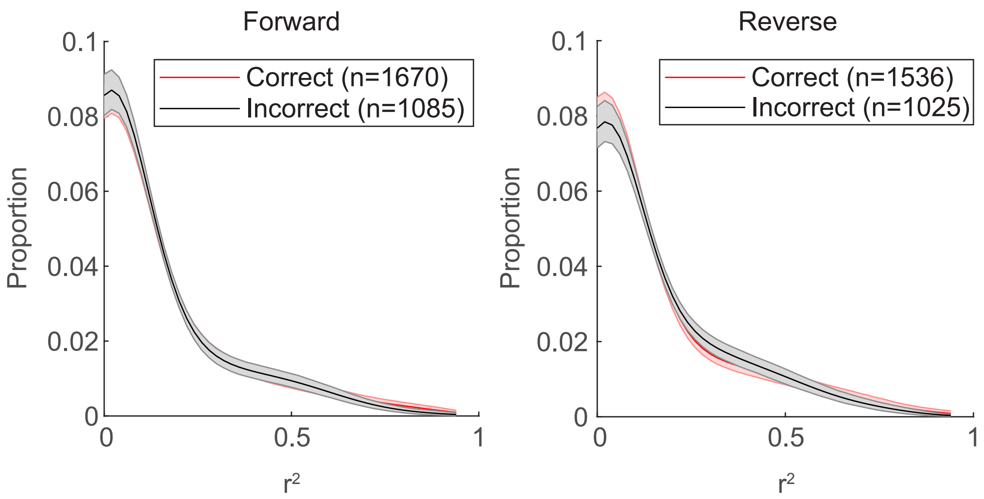

Supplementary Figure 8

Supplementary Figure 9. A bias for replay events to terminate at the correct reward location occurs in rest periods before and after the test phase during correct trials but not error trials.

a, Mean posterior probabilities of replay events in rest periods of correct trials before the test phase (left panel) and after the test phase (right panel). Replay events were aligned to the correct goal location on the y-axis (i.e., location 0). Normalized time of replay event is shown on the x-axis (i.e., replay offset at time = 1). **b**, The sum of posterior probability for the last normalized time bin across replay events for each location number relative to the correct reward location. Dashed black lines mark 95% confidence intervals of a null distribution generated by randomly shifting positions of each replay event, as in Fig. 7b and d. Red bars mark the location numbers with a posterior probability sum that exceeded the corresponding 95% confidence interval (i.e., location 0, which corresponds to the correct reward location). **c**, Same as **a** but for error trials. **d,** Same as **b** but for error trials. In this panel, no bars are red because none of the locations' summed posterior probabilities exceeded the 95% confidence intervals for error trials.

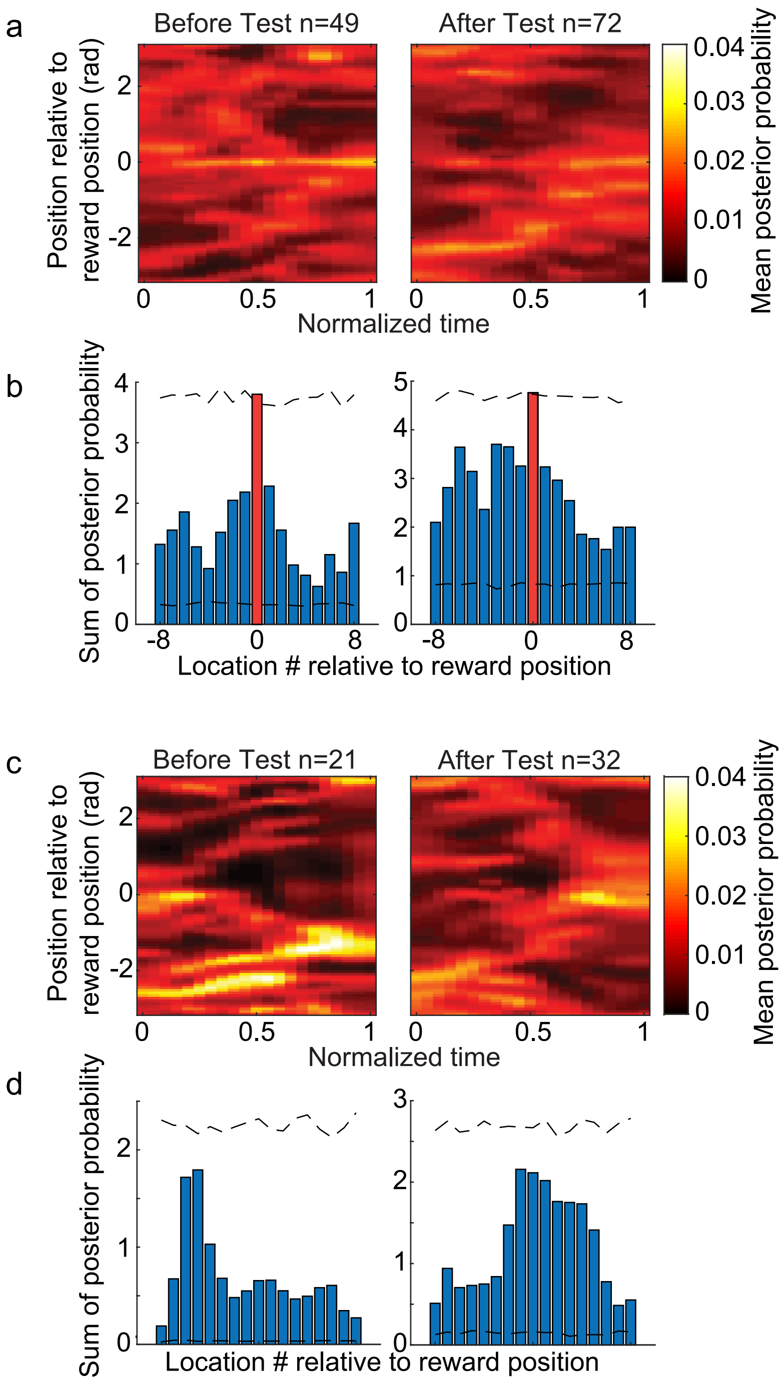

Supplementary Figure 9

Supplementary Figure 10. A significant bias for replay events to terminate at the correct reward location did not occur in post-test trials and pre-running trials.

a, Mean posterior probabilities of replay events in post-test trials. Replay events were aligned to the correct reward location on the vertical axis (i.e., location 0). Normalized time of replay event is shown on the horizontal axis (i.e., replay offset at time = 1). Correct and error post-test trials are shown in the left and right panels, respectively. **b**, The sum of posterior probability for the last normalized time bin across replay events for each location number relative to the goal. Dashed black lines mark 95% confidence intervals of a null distribution generated by randomly shifting positions of each replay event, as in Fig. 7b and d. **c-d**, Same as **a-b** but for pre-running trials. Since there was no memory test or reward in pre-running trials, all trials were of the same type (i.e., no correct and error trials).

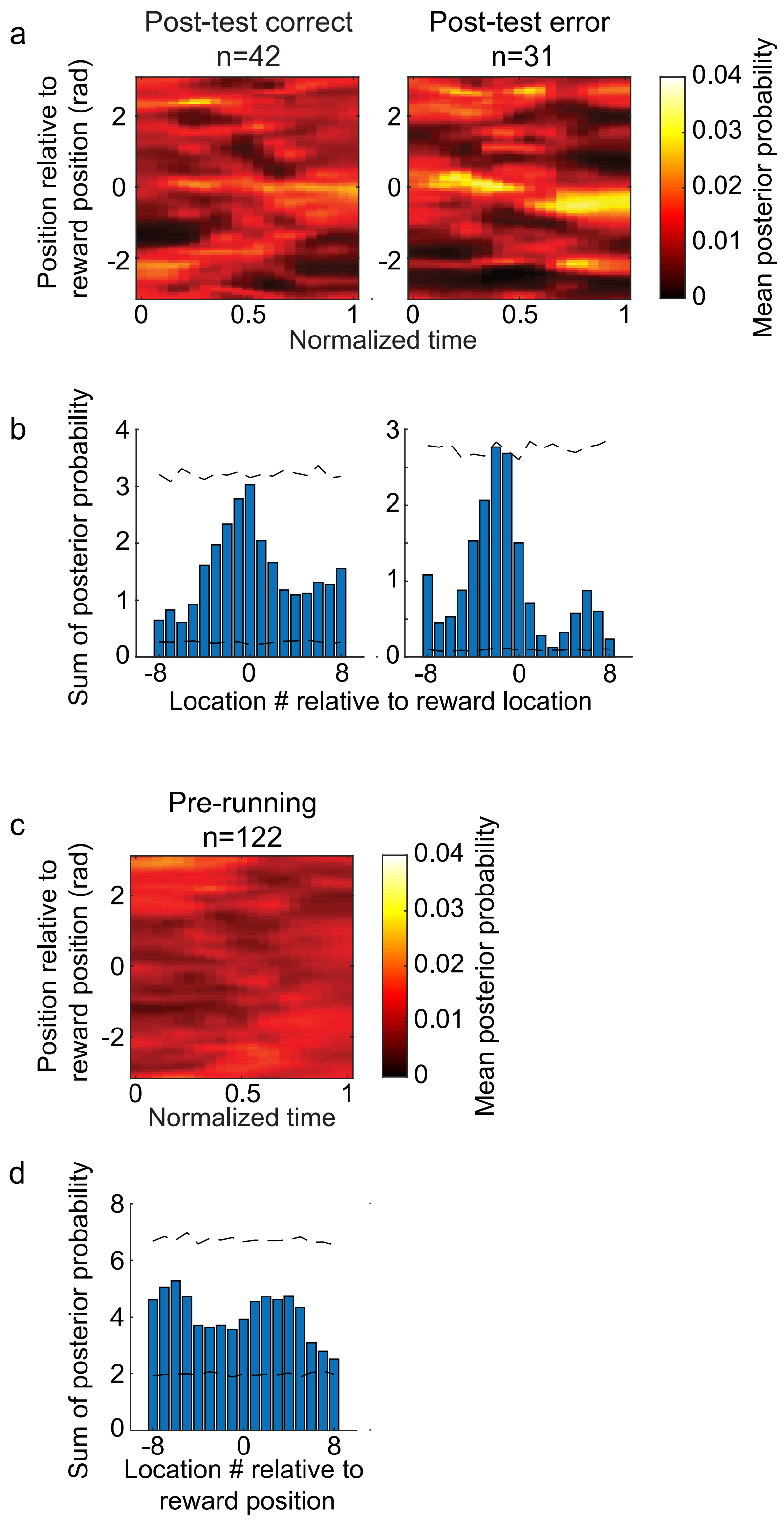

Supplementary Figure 10

Supplementary Fig. 11. No bias for replay events to start at the correct reward location was observed.

a, Mean posterior probabilities of replay events in correct trials 1-4 (left panel) and correct trials 5-8 (right panel). Replay events were aligned to the correct reward location on the y-axis (i.e., location 0). Normalized time is shown on the x-axis (replay onset at time = 0). Note that these posterior probability distributions are identical to those shown in Figure 7a. **b**, The sum of posterior probability for the first normalized time bin across replay events is shown for each location number relative to the correct reward location (i.e., location 0). Dashed black lines mark 95% confidence intervals of a null distribution generated by randomly shifting positions of each replay event. None of the locations' summed posterior probabilities exceeded the 95% confidence intervals. **c-d**, Same as **a-b** but for error trials. Note that the posterior probability distributions in panel c are identical to those shown in Figure 7c.

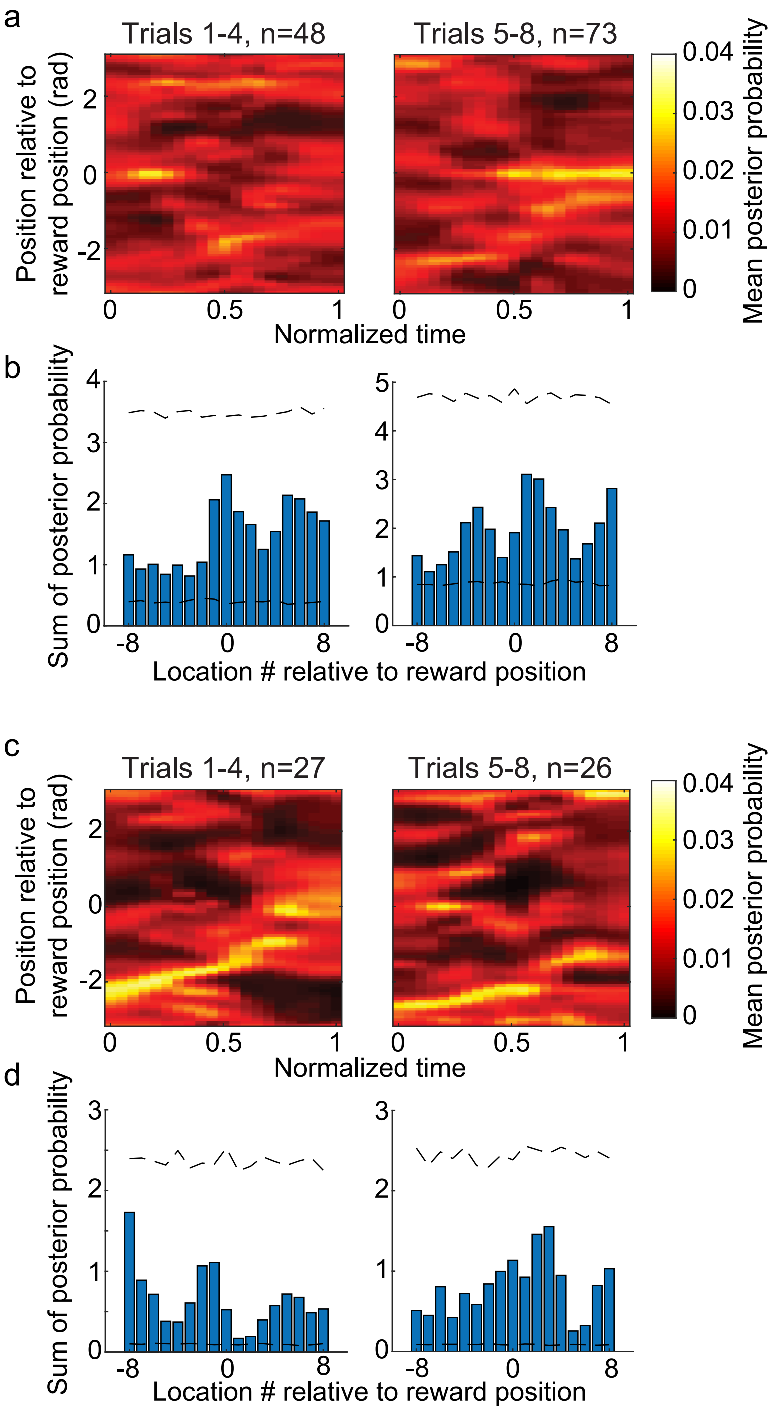

Supplementary Figure 11

SUPPLEMENTARY REFERENCES

1. Zheng, C., Bieri, K. W., Hsiao, Y. T. & Colgin, L. L. Spatial Sequence Coding Differs during Slow and Fast Gamma Rhythms in the Hippocampus. Neuron 89, 398–408 (2016).
