# Supplementary material for: Hippocampal place cell sequences differ during correct and error trials in a spatial memory task
Source: Nat Commun. 2021 Jun 7;12:3373. doi: 10.1038/s41467-021-23765-x (PMC8185092; doi:10.1038/s41467-021-23765-x)
Supplement: Supplementary file 3 — Reporting summary [file 41467_2021_23765_MOESM3_ESM.pdf]

nature research

Corresponding author(s): Chenguang Zheng & Laura Lee Colgin

Last updated by author(s): 5/6/2021

Reporting Summary

Nature Research wishes to improve the reproducibility of the work that we publish. This form provides structure for consistency and transparency in reporting. For further information on Nature Research policies, see Authors & Referees and the Editorial Policy Checklist .

Statistics

For all statistical analyses, confirm that the following items are present in the figure legend, table legend, main text, or Methods section.

n/a	Confirmed	
☐	☒	The exact sample size (*n*) for each experimental group/condition, given as a discrete number and unit of measurement
☐	☒	A statement on whether measurements were taken from distinct samples or whether the same sample was measured repeatedly
☐	☒	The statistical test(s) used AND whether they are one- or two-sided<br>*Only common tests should be described solely by name; describe more complex techniques in the Methods section.*
☒	☐	A description of all covariates tested
☐	☒	A description of any assumptions or corrections, such as tests of normality and adjustment for multiple comparisons
☐	☒	A full description of the statistical parameters including central tendency (e.g. means) or other basic estimates (e.g. regression coefficient) AND variation (e.g. standard deviation) or associated estimates of uncertainty (e.g. confidence intervals)
☐	☒	For null hypothesis testing, the test statistic (e.g. *F*, *t*, *r*) with confidence intervals, effect sizes, degrees of freedom and *P* value noted<br>*Give P values as exact values whenever suitable.*
☐	☒	For Bayesian analysis, information on the choice of priors and Markov chain Monte Carlo settings
☒	☐	For hierarchical and complex designs, identification of the appropriate level for tests and full reporting of outcomes
☒	☐	Estimates of effect sizes (e.g. Cohen's *d*, Pearson's *r*), indicating how they were calculated

Our web collection on statistics for biologists contains articles on many of the points above.

Software and code

Policy information about availability of computer code

Data collection	Collection was done using Neuralynx Cheetah version 5.0
Data analysis	Single units were separated using the MClust v3.5 package for MATLAB 2014a. Analysis was performed using MATLAB. The behavioral learning curves in Figure 1 were calculated using “Software for Bayesian analysis of learning” (https://health.ucdavis.edu/anesthesiology/research/bayes/index.html), with scripts running in Winbugs (WinBUGS14) in Matlab. Bayesian decoding analyses were performed using custom routines in MATLAB that can be found on github.com/ColginLab. Circular statistics were performed using the CircStat Toolbox version 1.21.0.0 (https://www.mathworks.com/matlabcentral/fileexchange/10676-circular-statistics-toolbox-directional-statistics).

For manuscripts utilizing custom algorithms or software that are central to the research but not yet described in published literature, software must be made available to editors/reviewers. We strongly encourage code deposition in a community repository (e.g. GitHub). See the Nature Research guidelines for submitting code & software for further information.

Data

Policy information about availability of data

All manuscripts must include a data availability statement. This statement should provide the following information, where applicable:

- Accession codes, unique identifiers, or web links for publicly available datasets
- A list of figures that have associated raw data
- A description of any restrictions on data availability

The datasets used in this study are available for download from https://web.corral.tacc.utexas.edu/Colgin_Nature_Comms_2021/. There are no restrictions on data availability.

Field-specific reporting

Please select the one below that is the best fit for your research. If you are not sure, read the appropriate sections before making your selection.

[x] Life sciences   [ ] Behavioural & social sciences   [ ] Ecological, evolutionary & environmental sciences

For a reference copy of the document with all sections, see nature.com/documents/nr-reporting-summary-flat.pdf

Life sciences study design

All studies must disclose on these points even when the disclosure is negative.

Sample size	Number of subjects and cells were selected to be on par with other papers studying place cell sequences (e.g., 4 rats used in Wikenheiser and Redish, Nature Neuroscience 2015, doi:10.1038/nn.3909; 4 rats used in Wang, Foster, and Pfeiffer, Science 2020, DOI: 10.1126/science.abb4151).
Data exclusions	Place cells with peak firing rates above 0.5 Hz at a goal location after pre-running were randomly downsampled to match the number of place cells with peak firing rates above 0.5 Hz elsewhere on the track, excluding the positions that corresponded to the rest box. This procedure was done to ensure that our Bayesian decoder was not biased by proportion of active place cells at the goal location on the track.
Replication	Analyses done in this study were verified by two independent experimenters. Results were not replicated with a second set of rats because of the time and difficulty involved in obtaining large ensembles of place cells in freely behaving rats. This study took years to complete. Please note that other studies involving recordings of large ensembles of place cells and decoding analyses also did not replicate findings in a second set of rats (e.g., Wikenheiser and Redish, Nature Neuroscience 2015; Wang, Foster, and Pfeiffer, Science 2020).
Randomization	The experimental design of this study did not involve any subject grouping, and thus group randomization was not applicable.
Blinding	The experimental design of this study did not involve any subject grouping, and thus data collection and analyses were not performed blind.

Reporting for specific materials, systems and methods

We require information from authors about some types of materials, experimental systems and methods used in many studies. Here, indicate whether each material, system or method listed is relevant to your study. If you are not sure if a list item applies to your research, read the appropriate section before selecting a response.

Materials & experimental systems

n/a	Involved in the study	
[x]	[ ]	Antibodies
[x]	[ ]	Eukaryotic cell lines
[x]	[ ]	Palaeontology
[ ]	[x]	Animals and other organisms
[x]	[ ]	Human research participants
[x]	[ ]	Clinical data

Methods

n/a	Involved in the study	
[x]	[ ]	ChIP-seq
[x]	[ ]	Flow cytometry
[x]	[ ]	MRI-based neuroimaging

Animals and other organisms

Policy information about studies involving animals; ARRIVE guidelines recommended for reporting animal research

Laboratory animals	Male Long-Evans rats weighing from ~400 to 600 g were used in this study. Their ages ranged from 4 to 12 months old.
Wild animals	This study did not involve wild animals.
Field-collected samples	This study did not involved field-collected samples.
Ethics oversight	All experiments were conducted according to the guidelines of the United States National Institutes of Health Guide for the Care and Use of Laboratory Animals under a protocol approved by the University of Texas at Austin Institutional Animal Care and Use Committee.

Note that full information on the approval of the study protocol must also be provided in the manuscript.
